# Online Porn Addiction: What We Know and What We Don’t—A Systematic Review

**DOI:** 10.3390/jcm8010091

**Published:** 2019-01-15

**Authors:** Rubén de Alarcón, Javier I. de la Iglesia, Nerea M. Casado, Angel L. Montejo

**Affiliations:** 1 Psychiatry Service, Hospital Clínico Universitario de Salamanca, Institute of Biomedical Research of Salamanca (IBSAL), 37007 Salamanca, Spain; ruperghost@gmail.com (R.d.A.); javidelaiglesia.jdli@gmail.com (J.I.d.l.I.); nmcasado91@gmail.com (N.M.C.); 2 University of Salamanca, EUEF, 37007 Salamanca, Spain

**Keywords:** online pornography, addiction, cybersex, internet, compulsive sexual behavior, hypersexuality

## Abstract

In the last few years, there has been a wave of articles related to behavioral addictions; some of them have a focus on online pornography addiction. However, despite all efforts, we are still unable to profile when engaging in this behavior becomes pathological. Common problems include: sample bias, the search for diagnostic instrumentals, opposing approximations to the matter, and the fact that this entity may be encompassed inside a greater pathology (i.e., sex addiction) that may present itself with very diverse symptomatology. Behavioral addictions form a largely unexplored field of study, and usually exhibit a problematic consumption model: loss of control, impairment, and risky use. Hypersexual disorder fits this model and may be composed of several sexual behaviors, like problematic use of online pornography (POPU). Online pornography use is on the rise, with a potential for addiction considering the “triple A” influence (accessibility, affordability, anonymity). This problematic use might have adverse effects in sexual development and sexual functioning, especially among the young population. We aim to gather existing knowledge on problematic online pornography use as a pathological entity. Here we try to summarize what we know about this entity and outline some areas worthy of further research.

## 1. Introduction

With the inclusion of “Gambling Disorder” in the “Substance Use and Addictive Disorders” chapter of the DSM-5 [1], the APA publicly acknowledged the phenomenon of behavioral addiction. Furthermore, “Internet Gaming Disorder” was placed in Section 3—conditions for further study.

This represents the ongoing paradigm shift in the field of addictions that relates to addictive behavior, and paves the way for new research in the light of cultural changes caused by the new technologies. 

There is apparently an existing common neurobiological [2] and environmental [3] ground between the varying addictive disorders, including both substance abuse and addictive behavior; this can manifest as an overlapping of both entities [4].

Phenomenologically, behaviorally addicted individuals frequently exhibit a problematic consumption model: impaired control (e.g., craving, unsuccessful attempts to reduce the behavior), impairment (e.g., narrowing of interests, neglect of other areas of life), and risky use (persisting intake despite awareness of damaging psychological effects). Whether these behaviors also meet physiological criteria relating to addiction (tolerance, withdrawal) is more debatable [4,5,6]. 

Hypersexual disorder is sometimes considered one of those behavioral addictions. It is used as an umbrella construct that encompasses various problematic behaviors (excessive masturbation, cybersex, pornography use, telephone sex, sexual behavior with consenting adults, strip club visitations, etc.) [7]. Its prevalence rates range from 3% to 6%, though it is difficult to determine since there is not a formal definition of the disorder [8,9]. 

The lack of robust scientific data makes its research, conceptualization, and assessment difficult, leading to a variety of proposals to explain it, but is usually associated with significant distress, feelings of shame and psychosocial dysfunction [8], as well as other addictive behaviors [10] and it warrants direct examination. 

Concurrently, the rise of the new technologies has also opened up a pool of problematic addictive behavior, mainly Internet Addiction. This addiction may focus on a specific application on the internet (gaming, shopping, betting, cybersex…) [11] with potential for risk-addictive behavior; in this case, it would act as a channel for concrete manifestations of said behavior [4,12]. This means inevitable escalation, providing new outlets for established addicts as well as tempting people (due to increased privacy, or opportunity) who would not have previously engaged in these behaviors.

Online pornography use, also known as Internet pornography use or cybersex, may be one of those Internet-specific behaviors with a risk for addiction. It corresponds to the use of Internet to engage in various gratifying sexual activities [13], among which stands the use of pornography [13,14] which is the most popular activity [15,16,17] with an infinite number of sexual scenarios accessible [13,18,19,20]. Continued use in this fashion sometimes derives in financial, legal, occupational, and relationship trouble [6,21] or personal problems, with diverse negative consequences. Feelings of loss of control and persistent use despite these adverse results constitute “online sexual compulsivity” [22] or Problematic Online Pornography Use (POPU). This problematic consumption model benefits from the “Triple A” factors [23].

Due to this model, pornography-related masturbation may be more frequent nowadays, but this is not necessarily a sign of pathology [21]. We know that a considerable proportion of young male population access Internet for pornography consumption [24,25]; in fact, it is one of their key sources for sexual health [26]. Some have expressed concern about this, addressing the time gap between when porn material is consumed for the first time ever, and an actual first sexual experience; specifically, how the former can have an impact on sexual development [27] like abnormally low sexual desire when consuming online pornography [28] and erectile dysfunction, which has spiked dramatically among young men in the past few years when compared to a couple decades ago [29,30,31,32,33].

We systematically reviewed the existing literature on the subject of POPU to try and summarize the various recent advances made in terms of epidemiology, clinical manifestations, neurobiological evidence that supports this model of problematic use, its diagnostic conceptualization in relation to hypersexual disorder, its proposed assessment instruments and treatment strategies.

## 2. Methods

**Figure 1:**
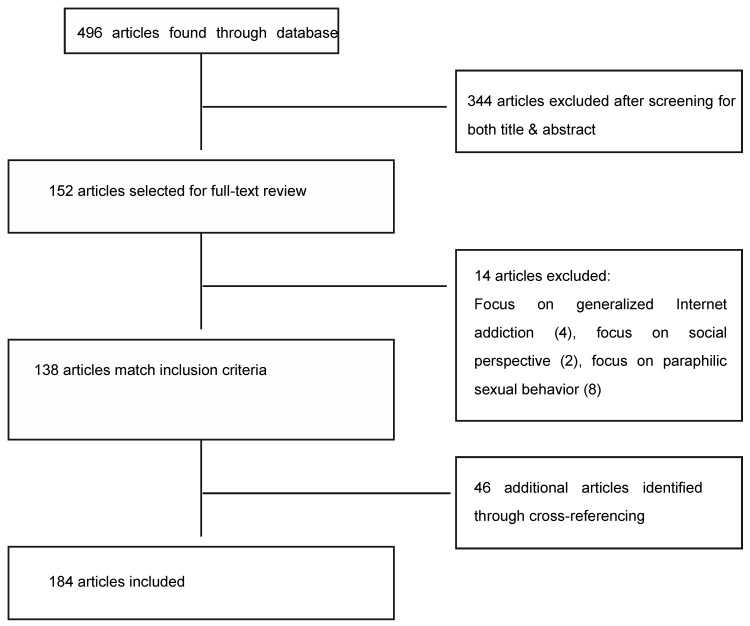
PRISMA flow diagram.

We performed the systematic review following PRISMA guidelines (Figure 1). Given the relatively new body of evidence regarding this subject, we conducted our review with no specific time-delimitation. Priority was placed upon literature reviews and articles published via a newest to oldest methodology, preferentially for already published reviews on the subject. PubMed and Cochrane were the main databases used, though a number of articles were compiled through cross-referencing.

Since our focus was mainly online pornography and addictive sexual behavior, we excluded those articles that had only a peripheral association with it in our search: those with a focus on generalized Internet addiction, those centered on the pornographic equivalent of varying paraphilias, and those that approached the subject from a social perspective.

The following search terms and their derivatives were used in multiple combinations: cybersex, porn* (to allow for both “pornography” and “pornographic”), addict* (to allow for both “addiction” and “addictive”), online, internet, sex, compulsive sex, hypersexuality. The reference management tool Zotero was used to build a database of all articles considered.

## 3. Results

### 3.1. Epidemiology

Pornography consumption in the general population proves difficult to be adequately measured, especially since the rise of the Internet and the “triple A” factors which have allowed for both privacy and ease of access. Wright’s study about the use of pornography in U.S. male population using the General Social Survey (GSS) [34], and Price’s study (which expands upon Wright’s by distinguishing among age, cohort, and period effects) [35] constitute some of the few, if not the only ones, existing sources that track pornography use in the general population. They show the overall increasing consumption of pornography over the years, especially among male population in contrast to females. This is particularly prevalent among young adults, and it steadily decreases with age. 

Some interesting facts about pornography consumption tendencies stand out. One of them is that the 1963 and 1972 male cohort showed only a very small decline on their usage from the year 1999 onwards, suggesting that porn consumption among these groups has remained relatively constant since [35]. The other one is that 1999 is also the year the tendency for women aged 18 to 26 to consume pornography became three times as likely than the ones aged 45 to 53, instead of just two times as likely as it used to be up until that point [35]. These two facts could be related to changing tendencies in pornography consumption motivated by technology (switching from the offline to the online model of consumption), but it is impossible to know for sure since the original data does not account for differences in both offline and online variants when tracking pornography usage.

As for POPU, there is no clear and reliable data in the literature reviewed that can offer a solid estimation of its prevalence. Adding up to the already mentioned motives for lack of data on general pornography consumption, part of it might stem from the perceived taboo nature of the topic at hand by possible participants, the wide range of assessment tools used by researchers, and the lack of consensus on what actually constitutes a pathological usage of pornography, which are all issues also reviewed further into this paper.

The vast majority of studies pertaining POPU or hypersexual behavior prevalence use convenience samples to measure it, usually finding, despite population differences, that very few users consider this habit an addiction, and even when they do, even fewer consider that this could have a negative effect on them. Some examples:

(1) A study assessing behavioral addictions among substance users, found that only 9.80% out of 51 participants considered they had an addiction to sex or pornography [36].
(2) A Swedish study that recruited a sample of 1913 participants through a web questionnaire, 7.6% reported some Internet sexual problem and 4.5% indicated feeling ‘addicted’ to Internet for love and sexual purposes, and that this was a ‘big problem’ [17].
(3) A Spanish study with a sample of 1557 college students found that 8.6% was in a potential risk of developing a pathological usage of online pornography, but that the actual pathological user prevalence was 0.7% [37].

The only study with a representative sample to date is an Australian one, with a sample of 20,094 participants; 1.2% of the women surveyed considered themselves addicted, whereas for the men it was 4.4% [38]. Similar findings also apply to hypersexual behavior outside of pornography [39].

Predictors for problematic sexual behavior and pornography use are, across populations: being a man, young age, religiousness, frequent Internet use, negative mood states, and being prone to sexual boredom, and novelty seeking [17,37,40,41]. Some of this risk factors are also shared by hypersexual behavior patients [39,42]. 

### 3.2. Ethiopathogenical and Diagnostic Conceptualization

Conceptualizing pathological behaviors continues to be a challenge today. While several attempts have been made regarding hypersexual behavior, the lack of robust data as of now explains the fact that there’s no consensus on this matter [9]. POPU comprises a very specific set of sexual behaviors that involve technology. Due to problematic technology use (especially online technology) being relatively recent, we need first to talk about hypersexual behavior not related to technology in order to understand the place of online pornography in it.

Sexuality as a behavior is vastly heterogeneous, and its potential pathological side has been studied for centuries [43]. Therefore, it represents a challenge to models trying to adequately define it, since it can incorporate practices ranging from solitary fantasizing to sexual violence [21]. It is also difficult to define what constitutes an actual dysfunction and manage to avoid the possible misuse of that definition to stigmatize and pathologize individuals [44]. For example, some set the limit between normal and pathological sexual behavior at more than seven orgasms in a week [43] (p. 381), but this approach focusing on quantity can be dangerous, since what constitutes normal and pathological behavior can vastly vary between individuals. This lack of uniformity and consistency in its classification may hinder future research on investigating hypersexual behavior [45] and ignore the quality aspects that focus on the negative emotions associated with it [46,47]. There have been proposals to redeem this issue using certain tools, already developed as part of the hypersexual disorder proposal used in the DSM-5 field trial [43,47]. 

Hypersexuality generally acts as an umbrella construct [7]. Its nomenclature is still a matter of debate to this day, and it is frequent to encounter several terms that refer to the same concept: compulsive sexual behavior, sex addiction, sexual impulsivity, hypersexual behavior or hypersexual disorder. Some authors, while recognizing the value of the terms “addiction” and “compulsivity”, prefer to draw attention to the issue of control and its possible loss or compromise as the primary concern about this behavior, thus referring to it as “out of control sexual behavior” [45,48,49].

Although definitions are not uniform, they usually focus on the frequency or intensity of symptoms [46] of otherwise normal urges and fantasies, that would result in dysfunction. This differentiates it from paraphilic sexual behavior, though the need for a better clarification of possible differences, similarities, and overlap between the two types still persists [45]. 

Usually included in hypersexual behavior are excessive masturbation and various sexual related behaviors, like dependence on anonymous sexual encounters, repetitive promiscuity, internet pornography, telephone sex, and visiting strip clubs [43,44,49,50,51]. Bancroft particularly thought that, in using Internet, both masturbation and these sexual activities could blend themselves, stating that men “use it as an almost limitless extension of their out of control masturbatory behavior”.

While the possibility to diagnose hypersexual behavior was always available with “sexual disorder not otherwise specified” in the DSM [1], Kafka [43] tried to propose it as a diagnostic entity for the DSM-5. He presented a set of criteria for it, as part of the sexual disorders chapter. These proposed models included hypersexual behavior as: (1) sexually motivated, (2) a behavioral addiction, (3) part of the obsessive-compulsive spectrum disorder, (4) part of the impulsivity-spectrum disorders, and (5) an “out of control” excessive sexual behavior. This proposal was ultimately rejected due to several reasons; the main was said to be absence of consolidated epidemiological and neuroimaging data regarding this behavior [52,53], but also its potential for forensic abuse, a not specific enough set of diagnostic criteria, and potential politic and social ramifications of pathologizing an integral area of behavior to human life [54]. It is interesting to compare it to the other two previous set of criteria present in the reviewed literature, those of Patrick Carnes and Aviel Goodman [9]. All three share the concepts of loss of control, excessive time spent on sexual behavior and negative consequences to self/others, but diverge on the other elements. This reflects in broad strokes the lack of consensus in conceptualizing hypersexual behavior across the years. Currently, the main options propose hypersexual behavior either as an impulse control disorder or a behavioral addiction [55]. 

From an impulse control disorder perspective, hypersexual behavior is generally referred to as Compulsive Sexual Behavior (CSB). Coleman [56] is a proponent of this theory. While he includes paraphilic behavior under this term [57], and they may coexist in some cases, he distinctly differentiates it from nonparaphilic CSB, which is what we want to focus on in this review. Interestingly, nonparaphilic hypersexual behavior is usually as frequent, if not more, than some paraphilias [43,58].

However, more recent definitions of CSB usually refer to multiple sexual behaviors that can be compulsive: the most commonly reported being masturbation, being followed by compulsive use of pornography, and promiscuity, compulsive cruising, and multiple relationships (22–76%) [9,59,60].

While there are definite overlaps between hypersexuality and conditions such as obsessive-compulsive disorder (OCD) and other impulse control disorders [61], there are also some notable differences pointed out: for example, OCD behaviors do not involve reward, unlike sexual behavior. Moreover, while engaging in compulsions might result in temporary relief for OCD patients [62], hypersexual behavior is usually associated by guilt and regret after committing the act [63]. Also, the impulsivity that can sometimes dominate the patient’s behavior is incompatible with the careful planning that is sometimes required in CSB (for example, in regards to a sexual encounter) [64]. Goodman thinks that addiction disorders lie at the intersection of compulsive disorders (which involve anxiety reduction) and impulsive disorders (which involve gratification), with the symptoms being underpinned by neurobiological mechanisms (serotoninergic, dopaminergic, noradrenergic, and opioid systems) [65]. Stein agrees with a model combining several ethiopathogenical mechanisms and proposes an A-B-C model (affective dysregulation, behavioral addiction, and cognitive dyscontrol) to study this entity [61].

From an addictive behavior standpoint, hypersexual behavior relies on sharing core aspects of addiction. These aspects, according to the DSM-5 [1], refer to the mentioned problematic consumption model applied to hypersexual behavior, both offline and online [6,66,67]. Evidence of tolerance and withdrawal in these patients might probably be key in characterizing this entity as an addictive disorder [45]. Problematic use of cybersex is also often conceptualized as a behavioral addiction [13,68].

The term “addiction” applying to this entity is still subject to great debate. Zitzman considers that the resistance to use the term addiction is “more a reflection of cultural sexual liberality and permissiveness than any lack of symptomatic and diagnostic correspondence with other forms of addiction” [69]. However, the term needs to be used with caution, since it can be interpreted as a justification for an irresponsible search for gratification and hedonist pleasure, and blame the disruptive consequences on it.

There has long been a debate between Patrick Carnes and Eli Coleman over the diagnostics of hypersexual behavior. Coleman has considered hypersexuality to be driven by the need to reduce some type of anxiety, not by sexual desire [56] having classified it in seven subtypes (one of them being use of online pornography) [57], while Carnes (who defined addiction as “a pathological relationship with a mood altering experience”) finds similitudes to other behavioral addictions like gambling, focusing on the loss of control and continued behavior despite negative consequences [70].

A thorough review of the literature by Kraus [71], concluded that despite these similitudes, significant gaps in the concept’s understanding complicate its classification as an addiction. The main concerns are aimed towards quantity of large-scale prevalence, longitudinal and clinical data (defining main symptoms and its diagnostic limits), supported by neuropsychological, neurobiological, and genetic data, as well as some information regarding possible treatment screening and prevention, and points to digital technology in hypersexual behavior as a key point for future research. 

The rise of the Internet increases the possibilities for sexual interactions, and not just online pornography (webcamming, casual sex websites). Even whether Internet use represents a conduit for other types of repetitive behavior (e.g., sexual behavior or gambling) or constitutes a different entity in its own right is still debated [72]. Nevertheless, if the case is the former, the previous evidence and considerations could very well apply to its online counterpart.

There is currently a need for empirically derived criteria that takes into account unique factors characterizing online (versus offline) sexual behaviors, since most of them do not have an offline version that can be compared to [73]. So far, there have been mentions of new phenomena when dealing with online sexual behavior, like the presence of online dissociation [74], which causes to “be mentally and emotionally detached when engaged, with compromised time and depersonalization”. This dissociation has already been described in relation to other online activities [75], which supports the notion that cybersex problematic use could be related to both internet and sex addiction [76].

Finally, we have to mention that a diagnostic entity called “compulsive sexual behavior disorder” is being included in the upcoming definitive edition of ICD-11, in the “impulse control disorders” chapter [77]. The definition can be consulted at https://icd.who.int/dev11/l-m/en#/http%3a%2f%2fid.who.int%2ficd%2fentity%2f1630268048.

The inclusion of this category in the ICD-11 may be a response to the relevance of this issue and attest to its clinical utility, whereas the growing but yet inconclusive data prevents us from properly categorizing it as a mental health disorder [72]. It is believed to provide a better tool (yet in refinement process) for addressing the needs of treatment seeking patients and the possible guilt associated [78], and also may reflect the ongoing debates regarding the most appropriate classification of CSB and its limited amount of data in some areas [55,71] (Table 1). This inclusion could be the first step towards recognizing this issue and expanding on it, one key point being undoubtedly its online pornography subtype. 

### 3.3. Clinical Manifestations

Clinical manifestations of POPU can be summed up in three key points:

- Erectile dysfunction: while some studies have found little evidence of the association between pornography use and sexual dysfunction [33], others propose that the rise in pornography use may be the key factor explaining the sharp rise in erectile dysfunction among young people [80]. In one study, 60% of patients who suffered sexual dysfunction with a real partner, characteristically did not have this problem with pornography [8]. Some argue that causation between pornography use and sexual dysfunction is difficult to establish, since true controls not exposed to pornography are rare to find [81] and have proposed a possible research design in this regard.
- Psychosexual dissatisfaction: pornography use has been associated with sexual dissatisfaction and sexual dysfunction, for both males and females [82], being more critical of one’s body or their partner’s, increased performance pressure and less actual sex [83], having more sexual partners and engaging in paid sex behavior [34]. This impact is especially noted in relationships when it is one sided [84], in a very similar way to marijuana use, sharing key factors like higher secrecy [85]. These studies are based on regular non-pathological pornography use, but online pornography may not have harmful effects by itself, only when it has become an addiction [24]. This can explain the relationship between the use of female-centric pornography and more positive outcomes for women [86].
- Comorbidities: hypersexual behavior has been associated with anxiety disorder, followed by mood disorder, substance use disorder and sexual dysfunction [87]. These findings also apply to POPU [88], also being associated with smoking, drinking alcohol or coffee, substance abuse [41] and problematic video-game use [89,90].

Having some very specific pornographic content interests has been associated with an increase in reported problems [17]. It has been debated if these clinical features are the consequence of direct cybersex abuse or due to the subjects actually perceiving themselves as addicts [91].

### 3.4. Neurobiological Evidence Supporting Addiction Model

Collecting evidence about POPU is an arduous process; main data on this subject is still limited by small sample sizes, solely male heterosexual samples and cross-sectional designs [71], with not enough neuroimaging and neuropsychological studies [4], probably due to conceptual, financial and logistic obstacles. In addition, while substance addiction can be observed and modeled in experimental animals, we cannot do this with a candidate behavioral addiction; this may limit our study of its neurobiological underpinnings [72]. Current knowledge gaps regarding the research of hypersexual behavior, as well as possible approaches for addressing them, are expertly covered and summarized in Kraus’ article [71]. Most of the studies found in our research pertain hypersexual behavior, with pornography being only one of its accounted accessories.

This evidence is based on an evolving understanding of the neural process among addiction-related neuroplasticity changes. Dopamine levels play an important part in this sexual reward stimuli, as observed already in frontotemporal dementia and pro-dopaminergic medication in Parkinson’s disease being linked with sexual behavior [92,93].

The addictive process with online pornography may be amplified by the accelerated novelty and the “supranormal stimulus” (term coined by Nobel prize winner Nikolaas Tinbergen) that constitutes Internet pornography [94]. This phenomenon would supposedly make artificial stimuli (in this case, pornography in the way it is mostly consumed today, its online form) override an evolutionarily developed genetic response. The theory is that they potentially activate our natural reward system at higher levels than what ancestors typically encountered as our brains evolved, making it liable to switch into an addictive mode [2]. If we consider online porn from this perspective, we can start seeing similarities to regular substance addicts.

Major brain changes observed across substance addicts lay the groundwork for the future research of addictive behaviors [95], including:

1. Sensitization [96]
2. Desensitization [97]
3. Dysfunctional prefrontal circuits (hypofrontality) [98]
4. Malfunctioning stress system [99]

These brain changes observed in addicts have been linked with patients with hypersexual behavior or pornography users through approximately 40 studies of different types: magnetic resonance imaging, electroencephalography (EEG), neuroendocrine, and neuropsychological.

For example, there are clear differences in brain activity between patients who have compulsive sexual behavior and controls, which mirror those of drug addicts. When exposed to sexual images, hypersexual subjects have shown differences between liking (in line with controls) and wanting (sexual desire), which was greater [8,100]. In other words, in these subjects there is more desire only for the specific sexual cue, but not generalized sexual desire. This points us to the sexual cue itself being then perceived as a reward [46].

Evidence of this neural activity signalizing desire is particularly prominent in the prefrontal cortex [101] and the amygdala [102,103], being evidence of sensitization. Activation in these brain regions is reminiscent of financial reward [104] and it may carry a similar impact. Moreover, there are higher EEG readings in these users, as well as the diminished desire for sex with a partner, but not for masturbation to pornography [105], something that reflects also on the difference in erection quality [8]. This can be considered a sign of desensitization. However, Steele’s study contains several methodological flaws to consider (subject heterogeneity, a lack of screening for mental disorders or addictions, the absence of a control group, and the use of questionnaires not validated for porn use) [106]. A study by Prause [107], this time with a control group, replicated these very findings. The role of cue reactivity and craving in the development of cybersex addiction have been corroborated in heterosexual female [108] and homosexual male samples [109].

This attentional bias to sexual cues is predominant in early hypersexual individuals [110], but a repeated exposure to them shows in turn desensitization [111,112]. This means a downregulation of reward systems, possibly mediated by the greater dorsal cingulate [107,113,114]. Since the dorsal cingulate is involved in anticipating rewards and responding to new events, a decrease in its activity after repeated exposure points us to the development of habituation to previous stimuli. This results in a dysfunctional enhanced preference for sexual novelty [115], which may manifest as attempts to overcome said habituation and desensitization through the search for more (new) pornography as a means of sexual satisfaction, choosing this behavior instead of actual sex [20].

These attempts at novelty seeking may be mediated through ventral striatal reactivity [116] and the amygdala [117]. It is known that the viewing of pornography in frequent users has also been associated with greater neural activity [99], especially in the ventral striatum [116,118] which plays a major role in anticipating rewards [119]. 

However, connectivity between ventral striatum and prefrontal cortex is decreased [103,113]; a decrease in connectivity between prefrontal cortex and the amygdala has also been observed [117]. In addition, hypersexual subjects have shown reduced functional connectivity between caudate and temporal cortex lobes, as well as gray matter deficit in these areas [120]. All of these alterations could explain the inability to control sexual behavior impulses.

Moreover, hypersexual subjects showed an increased volume of the amygdala [117], in contrast to those with a chronic exposure to a substance, which show a decreased amygdala volume [121]; this difference could be explained by the possible neurotoxic effect of the substance. In hypersexual subjects, increased activity and volume may reflect overlapping with addiction processes (particularly supporting incentive motivation theories) or be the consequence to chronic social stress mechanisms, such as the behavioral addiction itself [122].

These users also have shown a dysfunctional stress response, mainly mediated through the hypothalamus–pituitary–adrenal axis [122] in a way that mirror those alterations seen in substance addicts. These alterations may be the result of epigenetic changes on classic inflammatory mediators driving addictions, like corticotropin-releasing-factor (CRF) [123]. This epigenetic regulation hypothesis considers both hedonic and anhedonic behavioral outcomes are at least partially affected by dopaminergic genes, and possibly other candidate neurotransmitter-related gene polymorphisms [124]. There is also evidence of higher tumor necrosis factor (TNF) in sex addicts, with a strong correlation between TNF levels and high scores in hypersexuality rating scales [125].

### 3.5. Neuropsychological Evidence

In regard to the manifestations of these alterations in sexual behavior, most neuropsychological studies show some kind of indirect or direct consequence in executive function [126,127], possibly as a consequence of prefrontal cortex alterations [128]. When applied to online pornography, it contributes to its development and maintenance [129,130].

The specifics of this poorer executive functioning include: impulsivity [131,132], cognitive rigidity that impedes learning processes or the ability to shift attention [120,133,134], poor judgment and decision making [130,135], interference of working memory capacity [130], deficits in emotion regulation, and excessive preoccupation with sex [136]. These findings are reminiscent of other behavioral addictions (such as pathological gambling) and the behavior in substance dependencies [137]. Some studies directly contradict these findings [58], but there may be some limitations in methodology (for example, small sample size).

Approaching the factors that play a role in the development of hypersexual behavior and cybersex, there are a number of them. We can think of cue-reactivity, positive reinforcement and associative learning [104,109,136,138,139] as the core mechanisms of porn addiction development. However, there may be factors of underlying vulnerability [140], like: (1) the role of sexual gratification and dysfunctional coping in some predisposed individuals [40,141,142,143] whether it is a consequence of trait impulsivity [144,145] or state impulsivity [146], and (2) approach/avoidance tendencies [147,148,149]. 

### 3.6. Prognosis

Most of the studies referenced use subjects with a long-term exposure to online pornography [34,81,113,114], so its clinical manifestations appear to be a direct and proportional consequence of engaging in this maladaptive behavior. We mentioned difficulty in obtaining controls to establish causation, but some case reports suggest that reducing or abandoning this behavior may cause improvement in pornography-induced sexual dysfunction and psychosexual dissatisfaction [79,80] and even full recovery; this would imply that the previously mentioned brain alterations are somewhat reversible.

### 3.7. Assessment Tools

Several screening instruments exist for addressing CSB and POPU. They all rely on the responder’s honesty and integrity; perhaps even more so than regular psychiatry screening tests, since sexual practices are the most humbling due to their private nature.

For hypersexuality, there are over 20 screening questionnaires and clinical interviews. Some of the most notable include the Sexual Addiction Screening Test (SAST) proposed by Carnes [150], and its later revised version SAST-R [151], the Compulsive Sexual Behavior Inventory (CSBI) [152,153] and the Hypersexual Disorder Screening Inventory (HDSI) [154]. The HDSI was originally used for the clinical screening of the DSM-5 field proposal of hypersexual disorder. While further explorations of the empirical implications regarding criteria and the refinements of cutoff scores are needed, it currently holds the strongest psychometric support and is the best valid instrument in measuring hypersexual disorder [151].

As for online pornography, the most used screening tool is the Internet Sex-screening test (ISST) [155]. It assesses five distinct dimensions (online sexual compulsivity, online sexual behavior-social, online sexual behavior-isolated, online sexual spending and interest in online sexual behavior) through 25 dichotomic (yes/no) questions. However, its psychometric properties haven only been mildly analyzed, with a more robust validation in Spanish [156] that has served as a blueprint for posterior studies [157].

Other notable instruments are the problematic pornography use scale (PPUS) [158] which measures four facets of POPU (including: distress and functional problems, excessive use, control difficulties and use for escape/avoidance of negative emotions), the short internet addiction test adapted to online sexual activities (s-IAT-sex) [159], a 12-item questionnaire measuring two dimensions of POPU, and the cyber-pornography use inventory (CPUI-9) [160].

The CPUI-9 evaluates three dimensions: (1) access efforts, (2) perceived compulsivity, and (3) emotional distress. At first considered to have convincing psychometric properties [9], this inventory has more recently proved to be unreliable: the inclusion of the “emotional distress” dimension address levels of shame and guilt, which do not belong in an addiction assessment and thus skews the scores upward [161]. Applying the inventory without this dimension appears to accurately reflect to some extent compulsive pornography use. 

One of the most recent is the pornographic problematic consumption scale (PPCS) [162], based on Griffith six-component addiction model [163], though it does not measure addiction, only problematic use of pornography with strong psychometric properties. 

Other measures of POPU that are not designed to measure online pornography use but have been validated using online pornography users [9], include the Pornography Consumption Inventory (PCI) [164,165], the Compulsive Pornography Consumption Scale (CPCS) [166] and the Pornography Craving Questionnaire (PCQ) [167] which can assess contextual triggers among different types of pornography user. 

There are also tools for assessing pornography users’ readiness to abandon the behavior through self-initiated strategies [168] and an assessment of treatment outcome in doing so [169], identifying in particular three potential relapse motivations: (a) sexual arousal/boredom/opportunity, (b) intoxication/locations/easy access, and (c) negative emotions.

### 3.8. Treatment

Given that still many questions remain regarding the conceptualization, assessment, and causes of hypersexual behavior and POPU, there have been relatively few attempts to research possible treatment options. In published studies, sample sizes are usually small and too homogeneous, clinical controls are lacking, and the research methods are scattered, unverifiable, and not replicable [170]. 

Usually, combining psychosocial, cognitive–behavioral, psychodynamic, and pharmacologic methods is considered most efficient in treatment of sexual addiction, but this non-specific approach reflects the lack of knowledge about the subject [9].

#### 3.8.1. Pharmacological Approaches

The studies have centered on paroxetine and naltrexone thus far. One case series involving paroxetine on POPU helped to decrease the anxiety levels, but eventually failed to reduce the behavior by itself [171]. Additionally, using SSRIs to create sexual dysfunction through their side effects is apparently not effective, and according to clinical experience are useful only in patients with comorbid psychiatric disorders [172].

Four case reports involving naltrexone to treat POPU have been described. Previous findings have suggested that naltrexone could be a potential treatment for behavioral addictions and hypersexual disorder [173,174], theoretically reducing cravings and urges by blocking the euphoria associated with the behavior. While there is not yet a randomized controlled trial with naltrexone in these subjects, there are four case reports. Results obtained in reducing pornography use varied from good [175,176,177] to moderate [178]; at least in one of them the patient also received sertraline, so it is unclear how much can be attributed to naltrexone [176]. 

#### 3.8.2. Psychotherapeutic Approaches

Undoubtedly, psychotherapy can be an important tool in fully comprehending and changing a behavior. While cognitive-behavioral therapy (CBT) is considered by many clinicians to be useful in treating hypersexual disorder [179], a study that involved problematic online pornography users failed to achieve a reduction of the behavior [180], even if the severity of comorbid depressive symptoms and general quality of life was improved. This brings up the interest notion that merely reducing pornography use may not represent the most important treatment goal [170]. Other approaches using CBT to treat POPU have been made, but reoccurring methodological problems in this area prevent us from extracting reliable conclusions [181,182].

Psychodynamic psychotherapy and others like family therapy, couples’ therapy, and psychosocial treatments modeled after 12 step programs may prove vital when addressing themes of shame and guilt and restoring trust among the users’ closest relationships [170,172]. The only randomized controlled trial that exists with problematic online pornography users focuses on Acceptance and Commitment Therapy (ACT) [183], an improvement from their 2010 case series [184], which was the first experimental study to specifically address POPU. The study showed effective results but it is hard to extrapolate since the sample was again too small and focused on a very specific population. 

The reported success with CBT, conjoint therapy and ACT might rely on the fact that are based on mindfulness and acceptance frameworks; depending on the context, increasing pornography use acceptance may be equally or more important than reducing its use [170].

## 4. Discussion

It seems that POPU is not only one subtype of hypersexual disorder, but currently the most prevalent since it also frequently involves masturbation. Although this is difficult to accurately determine given the anonymity and accessibility factors that make pornography use today so pervasive, we can at least confirm that the patron of consumption for pornography has changed for roughly the last decade. It would not be absurd to assume its online variant has had a significant impact on its consumers, and that the triple A factors enhance the potential risk for POPU and other sexual behaviors.

As we mentioned, anonymity is a key risk factor for this sexual behavior to develop into a problem. We need to keep in mind that statistics regarding this problem are obviously limited to people of legal age to engage in sexual activity, online or otherwise; but it does not escape us that sexual activity rarely starts after this threshold, and there is a likely chance that minors still in the process of sexual neurodevelopment are a particularly vulnerable population. The truth is that a stronger consensus on what pathological sexual behavior constitutes, both offline and online, is necessary to adequately measure it in a representative manner and confirm how much of a problem it is in today’s society.

As far as we know, a number of recent studies support this entity as an addiction with important clinical manifestations such as sexual dysfunction and psychosexual dissatisfaction. Most of the existing work is based off on similar research done on substance addicts, based on the hypothesis of online pornography as a ‘supranormal stimulus’ akin to an actual substance that, through continued consumption, can spark an addictive disorder. However, concepts like tolerance and abstinence are not yet clearly established enough to merit the labeling of addiction, and thus constitute a crucial part of future research. For the moment, a diagnostic entity encompassing out of control sexual behavior has been included in the ICD-11 due to its current clinical relevance, and it will surely be of use to address patients with these symptoms that ask clinicians for help.

A variety of assessment tools exist to help the average clinician with diagnostic approaches, but delimiting what is truly pathological and not in accurate manner is still an ongoing problem. So far, a crucial part of the three sets of criteria proposed by Carnes, Goodman, and Kafka include core concepts of loss of control, excessive time spent on sexual behavior and negative consequences to self and others. In some manner or other, they are also present in the majority of screening tools reviewed.

They may be an adequate structure in which to build upon. Other elements, that are considered with varying degrees of importance, probably signal us to take individual factors into account. Devising an assessment tool that retains some level of flexibility while also being significant for determining what is problematic is surely another of the current challenges that we face, and will probably go in hand with further neurobiological research that help us better understand when a specific dimension of common human life shifts from normal behavior to a disorder. 

As for treatment strategies, the main goal currently focuses on reducing pornography consumption or abandoning it altogether, since clinical manifestations appear to be reversible. The way to achieve this varies accordingly to the patient and might also require some individual flexibility in the strategies utilized, with a mindfulness and acceptance-based psychotherapy being equally or more important than a pharmacological approach in some cases.

## Figures and Tables

**Table 1:** DSM-5 and ICD-11 approaches to classifying hypersexual behavior.

	DSM-5	ICD-11
Goal	Provide common research and clinical language for mental health problems	Reflect issues of clinical utility in a broad range of settings, global applicability, and scientific validity [79]
Conceptualization of hypersexual disorder	Addiction model	Impulse control model
Available diagnosis	No current hypersexual disorder diagnosis, due to insufficient evidence to categorize it as addiction	Compulsive sexual behavior disorder
